# Declining survival across invasion history for *Microstegium vimineum*

**DOI:** 10.1371/journal.pone.0183107

**Published:** 2017-08-15

**Authors:** Chelsea E. Cunard, Richard A. Lankau

**Affiliations:** 1 Department of Plant Biology, University of Georgia, Athens, Georgia, United States of America; 2 Plant Pathology Department, University of Wisconsin, Madison, Wisconsin, United States of America; Universidade da Coruna, SPAIN

## Abstract

Many alien species become invasive because they lack coevolutionary history with the native community; for instance, they may lack specialized enemies. These evolutionary advantages may allow the invader to establish and persist when rare within a community and lead to its monodominance through positive frequency dependence, i.e. increasing per capita population growth rate with increasing frequency of conspecifics. However, this advantage could degrade through time due to evolutionary and ecological changes in the invasive and native plant and microbial communities. We investigated survival rates and individual biomass as proxies for per capita population growth rates for the invasive grass, *Microstegium vimineum*, across a gradient of conspecific frequencies (10–100% relative cover of *M*. *vimineum*) within 12 sites that varied in time since invasion. We expected *M*. *vimineum* frequency dependence to become more negative and its proxies for population growth at low conspecific frequency to decline across invasion history. We also explored the belowground fungal community associated with *M*. *vimineum*, since we hypothesized that changes in *M*. *vimineum* population dynamics may result from shifting microbial interactions over time. *Microstegium vimineum* frequency dependence changed from negative to neutral across invasion history and the shift was driven by a decline in survival at low frequency. Changes in *M*. *vimineum* root fungal community were associated with time since invasion. Our results do not support a shift in frequency dependence from positive to negative across invasion history. However, our results suggest *M*. *vimineum* populations may be less prone to persist at older invaded sites and thus more vulnerable to management intervention.

## Introduction

Many alien species may gain an advantage over the native species due to a lack of coevolutionary history with the invaded community [1]. This evolutionary advantage could come in the form of novel weapons like plant allelochemicals [2–4], escape from specialized pests and pathogens [5–7], or unique niche requirements [8–10]. These advantages could allow invasive species to establish and then outcompete native species, and indeed invasive plants are commonly superior competitors [11] and are associated with decreases in diversity and abundance of native plants from local communities [12], even if they do not cause range wide native extinctions [13].

However, it remains unclear whether the dominance by invasive species will be stable over time or shift to coexistence between the invader and native species. A shift to coexistence could occur if the invasive species’ evolutionary advantage degrades through time as both the invasive and native species evolve post-introduction and/or ecological changes occur [14–16]. Specifically, pathogen accumulation could reduce this advantage [17] and larger pathogen loads or more negative net plant-soil microbial interactions have been found on plant species with older introduction dates [16, 18–20].

In order to establish in a new community, an introduced species must have a positive population growth rate when rare (i.e. at low conspecific frequency) [21, 22]. Furthermore, high per capita population growth rate when rare will act to buffer the invader’s population from local extinction, and promote spatial spread through the local area. On the other hand, if the invader’s per capita population growth rate is low (zero or negative), then the population will not be able to increase from its initial founding, and will be vulnerable to extinction when population density declines [22]. If high per capita population growth rates for invaders derive from evolutionary novelty, this effect may weaken over time as the invader accumulates greater interactions with community members, such as pests and pathogens. This reduction in growth rates could make the population more vulnerable to management intervention that reduces its frequency within a community.

While per capita population growth rate at low frequency determines whether an introduced species can establish, its ultimate relative abundance depends on frequency dependence. Whether an invasive plant can become monodominant or coexist with native plants depends on how its per capita population growth rate changes with conspecific frequency. An invasive plant that has positive frequency dependence (i.e. increasing per capita population growth rate with increasing frequency of conspecifics) would have the potential to form a monoculture. On the other hand, an invader with negative frequency dependence would have a population that equilibrates at a frequency where both natives and itself can persist, since per capita population growth rate decreases with increasing frequency of conspecifics. Since species interactions, especially with specialized enemies, are important sources of negative frequency dependent effects on host populations [22–25], evolutionary novelty may allow invaders to experience neutral or positive frequency dependence initially. However, if species interactions accumulate through time frequency dependence could become more negative.

The primary objective of this study was to investigate whether there was evidence for changes in frequency dependence and individual plant performance at low frequency (as relative proxies for per capita population growth rate in this annual species) for invasive *Microstegium vimineum* across invasion history. We predicted that *M*. *vimineum* frequency dependence would become more negative and performance at low frequency would decrease across time since invasion. We chose to study *M*. *vimineum* because it has invaded a wide area in eastern North America mainly through “natural” dispersal (i.e. not intentional planting), and has a simple annual lifestyle that allows a more direct link between individual performance and population dynamics.

The enemy release hypothesis has not been tested on *M*. *vimineum*, however aboveground accumulation of fungal pathogens that decrease *M*. *vimineum* performance have recently been reported in the invasive range [26]. In this study, we were specifically interested in the alteration of belowground interactions with soil microbes. Plant-soil feedbacks can become more negative with increasing time since invasion [16, 20] and this could affect the invader’s per capita population growth rate and frequency dependence. Therefore, our secondary objective was to identify any changes in the belowground fungal community that were correlated with invasion history and potentially affecting individual performance and frequency dependence of *M*. *vimineum*. We predicted that there would be changes in the fungal community directly associating with *M*. *vimineum* across invasion history.

## Materials and methods

### Study system

*Microstegium vimineum* is a C4, Asian annual grass. It was first collected within its invasive range in the United States near Knoxville, TN in 1919 [27]. It is a shade tolerant invader in eastern forests in the US [28, 29]. *Microstegium vimineum* reduces native plant biomass and diversity [30–32], suppresses tree seedling regeneration [33, 34], and is associated with changes in nutrient cycling and soil microbial communities [35–38]. Multiple mechanisms have been proposed for *M*. *vimineum* invasion success, including evolution post invasion [39], alteration in nutrient cycling [40, 41], and disturbance [42–44].

### Map of invasion history

**Fig 1:**
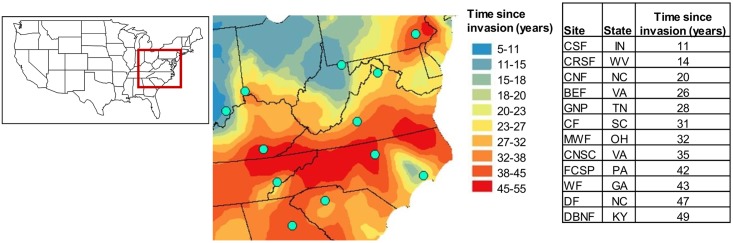
Map of *Microstegium vimineum* invasion history. Points on map represent the 12 sites where the study was performed and are also listed in the table with their abbreviated identification, state of location, and estimated time since invasion. Inset map of the United States depicts the location of the invasion history map.

We created a map of *M*. *vimineum* invasion history across the eastern United States using ArcMap in ArcGIS 10.1 (Fig 1). We created a database of *M*. *vimineum* collection dates and locations by contacting herbaria across the eastern US. We divided the eastern US into a 0.1 latitude by 0.1 longitude grid and assigned the oldest collection date to each cell, totaling 542 points. If a cell did not have an assigned date we left it blank. Using these data, we created a spatial kriging layer of invasion history.

We used the kriging function in ArcMap to interpolate estimated invasion ages based on the herbarium records. We used ordinary kriging, which assumes there is a constant trend between distance and the relationship between points, because we had no scientific explanation for using a model in which the trend varies across the landscape [45]. We used an exponential model to describe the spatial autocorrelation between points, which we determined as the model of best fit using the semivariogram [45, 46]. The kriging layer used the simplest model of invasion history, which excluded anisotropy (directional spatial autocorrelation), and had the appropriate lag size (43,500) and lag number (20), equaling about half of the largest distance between points (1,740,000 m) when multiplied together. The layer used the standard search neighborhood, with a maximum number of 5 points and a minimum number of 2 points within each of the 4 sectors that were at a 45° offset, to calculate predicted values from points.

To conclude whether the kriging layer was an appropriate interpolation surface for the data provided we explored its prediction errors. The average difference between the measured and predicted values was 0.0403 years, indicating that our predictions are relatively unbiased. The standardized mean prediction error was 0.0019, which is appropriately close to 0 [45]. The root mean squared standardized error was 0.9586 which is close to 1, meaning the prediction standard errors are valid. The root mean squared prediction error was 15.8590 and indicates how close the model predicts the actual point values. The average estimated prediction standard error was 16.6453. Since the root mean squared prediction error and the average estimated prediction standard error were similar and the root mean squared standardized error was close to 1, we concluded that the kriging layer was appropriate [45].

### Site and plot design

We then used the map to choose 12 sites that varied in *M*. *vimineum* time since invasion (Fig 1). The sites ranged from an estimated invasion age of approximately 11 to 49 years. These estimated invasion ages are most likely underestimates due to herbaria specimens’ collection dates more likely representing when a species becomes more abundant rather than initial establishment date. Exact invasion age estimates are difficult to acquire, but using herbaria collection data is excellent for comparative studies across large spatial and temporal gradients. We sampled at each of these sites twice, once at the end of May (spring) and then at the end of September (fall) 2012. At each site we established 8 1m^2^ plots in an area invaded by *M*. *vimineum*. We chose invaded areas for the study based on size—the area had to be at least 16m at its longest distance and at least 1m across. At sites CNF and BEF we were unable to locate invaded areas that met the size requirement, therefore we only established 4 plots at each of these sites. We randomly selected the plots along a transect of the invaded area. The transect went through the invaded area at its longest length and ranged from ~8m (at CNF and BEF) to ~150m. Plots varied from a minimum of 1m apart to a maximum of 18m apart. We randomly selected the first 6 plots and then, if necessary, intentionally chose the last 2 plots to try to maintain a range of percent cover of *M*. *vimineum*. We did this at each site so we could explore frequency dependence within sites. In the study, we had a total of 88 plots.

At all 8 plots at each site we measured variables that could potentially affect individual plant performance, frequency dependence, or be associated with time since invasion. We measured environmental variables, such as canopy openness and soil nutrients, to make sure any patterns we found in frequency dependence and individual plant performance across time since invasion were not confounded with other factors. At both sampling time points, within each plot we took percent cover estimates of 4 categories; grass/sedge, woody, herbaceous, and *M*. *vimineum*. We measured % canopy openness over each plot using hemispheric photographs taken with a digital camera (Canon EOS Rebel T3) with a fish eye lens (Opteka). The camera was held level 1m above the center of the plot. We analyzed the photos using Gap Light Analyzer software [47].

### Soil nutrients

During spring sampling, we collected soil from the top 10cm within each plot and oven dried it to analyze general nutrients (pH, P, K, Ca, Mg, MN, Zn) as well as total % nitrogen (N) and carbon (C) and the C:N ratio. We measured nitrate (NO_3_^-^) and ammonium (NH_4_^+^) in each plot by placing 20 g wet mass of mixed bed ion exchange resin (Rexyn^™^ 300 (H-OH) Beads (Analytical Grade/ Certified), Fisher Chemical) in the field for the duration of the study (~ 4 months). (see S1 Protocol for complete soil nutrient methods)

We condensed the 12 soil nutrient variables (pH, P, K, Ca, Mg, MN, Zn, N, C, C:N ratio, NO_3_^-^, NH_4_^+^) using a principal components analysis (PCA), the prcomp function, in R version 3.2.1 [48]. We used PCA axes 1, 2, and 3 in analyses, which explained 41.39%, 23.27%, and 13.19% of the total variation in the soil nutrient variables respectively, for a total of 77.85% of the variation explained. Total % N, Mn, Zn, and total % C had the highest loadings on PC1 of -0.4095, -0.4004, -0.3838, and -0.3784 respectively. K, Ca, Mg, and pH had the highest loadings on PC2 of 0.5790, 0.4719, 0.4443, and 0.3791 respectively. C:N ratio, NO_3_^-^, Mg, and Ca had the highest loadings on PC3 of 0.4749, 0.4531, -0.3747, and 0.3565 respectively. (see S1 Table for the loadings of the 12 soil variables onto PCA axes 1–12).

### Root fungal community

During the spring sampling, we collected *M*. *vimineum* roots from each plot to perform terminal restriction fragment length polymorphism analysis (T-RFLP) of the general fungal and arbuscular mycorrhizal fungal (AMF) community (see S1 Protocol for complete root fungal community methods).

### Estimating frequency dependence

During the spring sampling, we randomly tagged 5 *M*. *vimineum* individuals in each plot (n = 88) by zip tying them to metal stakes. In the fall sampling, we collected the aboveground biomass of any of the 5 individuals present, oven dried them at 60°C, and weighed their final biomass. Individuals that were gone from their marked spot were recorded as dead. We calculated the proportion of survivors as the number of live plants recovered at the end of the experiment divided by the total number of originally marked plants (5), and the average individual biomass of just live plants (g) as the total biomass of all recovered plants divided by the number of recovered plants. There is some possibility that plants that died before the fall sampling may have produced some seed, but this is probably very rare since *M*. *vimineum* usually starts to produce seeds in the fall (late September/early October) [49, 50]. Six plots were not relocated and are thus excluded from our analyses. The frequency of *M*. *vimineum* within each plot was calculated by dividing *M*. *vimineum* % cover by the total % cover of all plants, since % cover is a common, non-destructive metric used to estimate frequency and population dynamics [51]. We chose to explore both survival and individual biomass as proxies for per capita growth rate since both represent vital rates (survival, fecundity) that can have key roles in the population dynamics of an annual plant. Individual biomass is a reasonable predictor of seed production in this species (n = 68, R^2^ = 0.6668, unpublished data).

### Statistical analysis

#### Is frequency dependence associated with time since invasion?

To test whether *M*. *vimineum* frequency dependence changed with time since invasion, we regressed the proportion of survivors or the average living biomass of *M*. *vimineum* individuals within each plot against *M*. *vimineum* frequency, time since invasion, and their interaction and used site as a random effect and bootstrapped 95% confidence intervals (CI). Since frequency dependence is measured by the change in per capita fitness with increasing con-specific frequency in a plot, changes in frequency dependence across time since invasion would be depicted by a significant interaction between *M*. *vimineum* frequency and time since invasion. We ran a generalized linear mixed model with a binomial distribution when proportion of survivors was the dependent variable (n = 82) and a linear mixed model when it was average living biomass (n = 74). If we found a significant interaction between frequency and time since invasion within a model, we also used the estimate of the time since invasion main effect to understand how survival/average biomass at low frequency was associated with time since invasion, since the regression estimate reflects how time since invasion is associated with the variable at an *M*. *vimineum* frequency of 0.

A significant interaction between frequency and time since invasion could result from several distinct patterns: 1) a change in survival/biomass at high, but not low, frequency, 2) a change at low, but not high, frequency, or 3) opposite changes at high vs. low frequency. To examine which pattern drove a significant frequency by invasion history interaction, as well as to directly explore plant performance at low frequency across time since invasion, we used generalized linear models and likelihood ratio tests (LRT) to test whether *M*. *vimineum* survival changed across time since invasion in only high frequency or only low frequency plots (n = 12 for each subset). We chose the highest and lowest frequency plots from each site. For high frequency plots, frequency ranged from 0.55 to 0.90 in the spring, and 0.86 to 1 in the fall. For low frequency plots, frequency ranged from 0.10 to 0.43 in the spring and 0.11 to 0.77 in the fall. The four models, either using the highest or lowest frequency plots in spring or fall, included *M*. *vimineum* frequency as a covariate, since this varied among sites even in these subsets.

To visualize the changes in the relationship between *M*. *vimineum* survival and frequency across time since invasion we created a contour plot. We used a generalized linear model with a binomial distribution and proportion of survivors as the dependent variable with time since invasion, *M*. *vimineum* frequency, and their interaction as explanatory variables to create the response surface.

#### Is frequency dependence associated with other environmental variables?

To be confident that the pattern of frequency dependence changing across time since invasion, shown by a significant interaction between frequency and time since invasion for per capita survival, was not confounded with environmental variables, we used forward and backward stepwise regression and Akaike information criterion (AIC) to examine whether frequency dependence was changing across time since invasion, latitude (as a proxy for climatic gradients), soil nutrient PC axes (1–3), and % canopy openness. We focused on survival data only, since patterns across frequencies appeared to be driven primarily by differences in survival rather than growth of surviving plants. For our full model we regressed the proportion of survivors within each plot against *M*. *vimineum* frequency, each variable, and their interaction (n = 77), using a generalized linear model with a binomial distribution. We calculated the correlation coefficient (r) between each pair of variables used in the full model to make sure no variables were highly correlated (r > 0.5) We then explored the estimates of the parameters maintained in the final regression model.

As before, we analyzed high and low frequency subsets to further understand any patterns in the relationship between survival and frequency (frequency dependence) across environmental gradients (latitude, soil nutrient PC axes, % canopy openness). We used generalized linear models and LRTs to test whether *M*. *vimineum* survival changed across any environmental variables in only high frequency or only low frequency plots (n = 12 for each subset, except for soil nutrient PC axes n = 11). Because frequency still varied among sites within these two subsets, all models included *M*. *vimineum* frequency as a covariate.

All the above statistical analyses were ran with both spring and fall frequency estimates, since we were unsure which would have a larger effect on *M*. *vimineum* per capita population growth rate. Results for both frequency estimates were similar, therefore we only present our results using the spring frequency estimates.

#### Are changes in the fungal/AMF community correlated with time since invasion?

Using a permutational MANOVA through the adonis function in the vegan statistical package in R version 3.2.1, we tested whether changes in the root general fungal/AMF communities were associated with time since invasion. The fungal community operational taxonomic units’ (OTU) proportional abundances for each plot were averaged for each site to run these analyses (n = 12). If a fungal community was significantly associated with time since invasion we also tested whether it was associated with latitude, soil nutrient PC axes 1–3, and % canopy openness. If the community was significantly associated with any environmental variable as well as time since invasion we then included both factors in a model to be confident that the relationship between fungal community composition and time since invasion was not confounded with other factors.

## Results

### Is frequency dependence associated with time since invasion?

**Fig 2:**
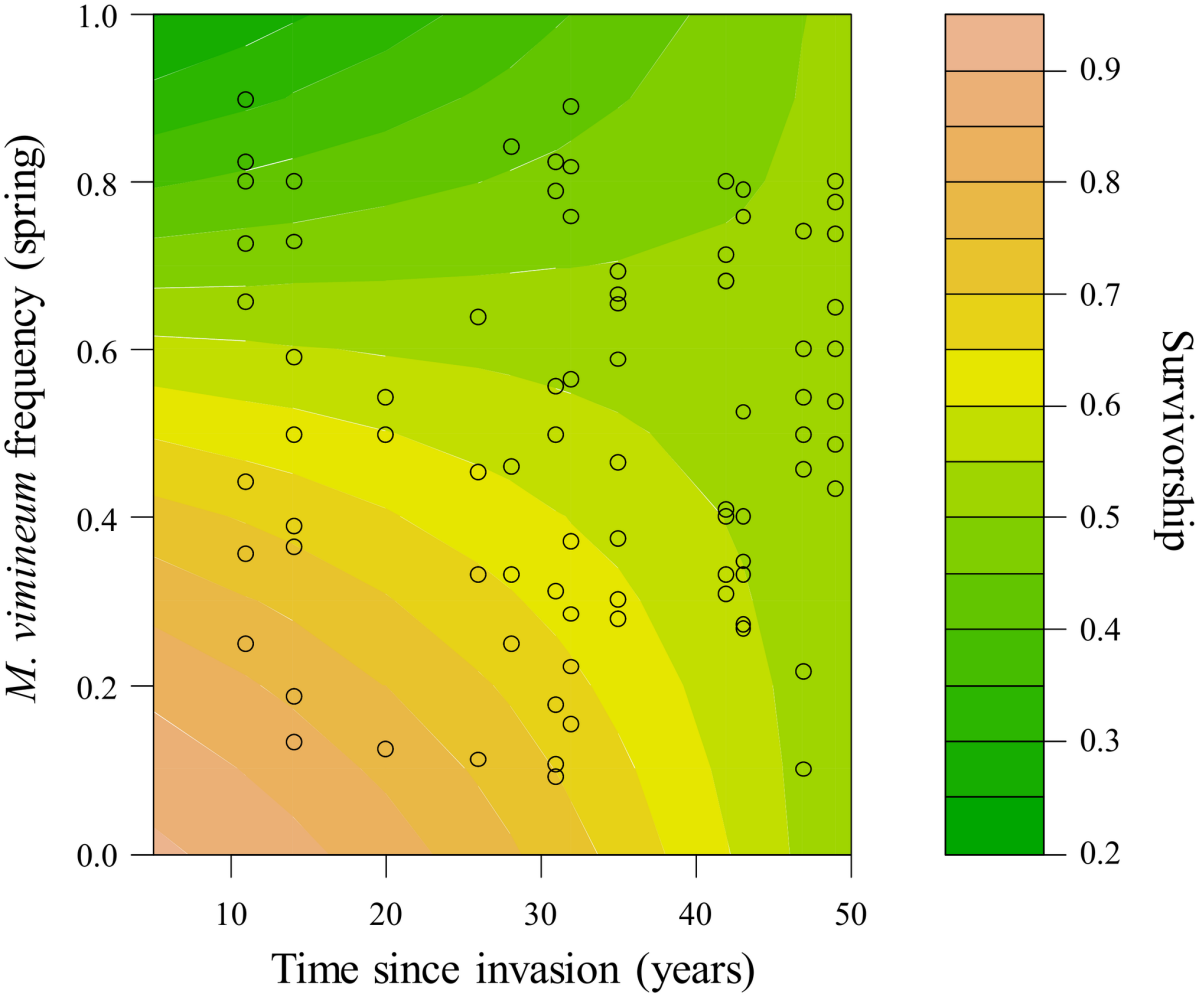
Survival across time since invasion and *Microstegium vimineum* frequency. Contour plot of proportion of *Microstegium vimineum* survivors across *M*. *vimineum* spring frequency and time since invasion of each of the 12 sites (n = 82).

Frequency dependence changed with time since invasion when the proportion of survivors was used as the dependent variable, shown by a 95% CI that did not overlap 0 for the interaction between *M*. *vimineum* frequency and time since invasion (CI = 0.0281–0.2261, Table 1). Survival decreased with increasing frequency of *M*. *vimineum* at younger invaded sites and was similar across all frequencies at older invaded sites (Fig 2), due to a decline in survival at low frequency across invasion time. This pattern is evident in the negative estimate for the main effect of time since invasion (-0.0738, Table 1), which in our models reflects the effect of invasion history at low frequency. Additionally, when we subsetted the data to include only high or low frequency plots, survival decreased across time since invasion at low *M*. *vimineum* frequency (LRT = 6.0360, P = 0.0140, S2 Table), but there was no such relationship across high frequency plots (S2 Table). The interaction between *M*. *vimineum* frequency and time since invasion had a 95% CI that overlapped 0 when we used average living biomass as the dependent variable (CI = -0.0088–0.0132, Table 1).

**Table 1:** Statistical results for mixed models testing per capita population growth rate metrics across time since invasion, *Microstegium vimineum* frequency, and their interaction.

Dependent variable	model term	estimate	standard error	95% CI: 2.50%	95% CI: 97.50%
proportion of survivors	time	-0.0738	0.0314	-0.1478	-0.0166
	frequency	-5.5092	1.5662	-8.8901	-2.6262
	time*freq	0.1195	0.0463	0.0281	0.2261
avg. individual biomass of live plants	time	-0.0042	0.0040	-0.0118	0.0040
	frequency	-0.0489	0.1712	-0.3680	0.3061
	time*freq	0.0026	0.0052	-0.0088	0.0132

Statistics for general liner mixed model with probability of *Microstegium vimineum* survival as the dependent variable (n = 82) and for linear mixed model with average individual biomass of *M*. *vimineum* live plants as the dependent variable (n = 74). 95% confidence intervals (CI) were obtained through bootstrapping. Time = time since invasion, frequency/freq = frequency of *M*. *vimineum* within a plot. Site was included as a random effect in each model.

### Is frequency dependence associated with other environmental variables?

The correlation coefficients ranged from 0.00–0.38 for the variables included in the full model for the stepwise AIC (S3 Table). When we included time since invasion, latitude, soil nutrient PC axes 1–3, % canopy openness and their interactions with *M*. *vimineum* frequency in a full model with survival as the dependent variable (AIC = 251.16), the final model with the lowest AIC (AIC = 244.14) included *M*. *vimineum* frequency, time since invasion, latitude, PC axes 1–3, and the interaction between *M*. *vimineum* frequency and latitude/PC axis 1 (Table 2). When we subsetted the data, survival declined across latitude and PC axes 2 and 3 and increased across PC axis 1 at high *M*. *vimineum* frequency (S2 Table). Survival at low *M*. *vimineum* frequency increased across % canopy openness (S2 Table).

**Table 2:** The final model terms of the model with the lowest AIC value.

Final model term	Estimate	Standard error	p-value (Wald test)
*M*. *vimineum* freq	24.1846	9.8796	0.0144
time since invasion	-0.0340	0.0110	0.0021
latitude	0.5693	0.1487	0.0001
soil nutrient PC1	0.0115	0.1398	0.9346
soil nutrient PC2	-0.3462	0.0823	2.62e-05
soil nutrient PC3	-0.3239	0.1021	0.0015
freq * latitude	-0.7063	0.2652	0.0077
freq * PC1	0.3999	0.2662	0.1331

The final model terms from the model with the lowest AIC value after stepwise regression was performed on a full model (n = 77). The probability of *M*. *vimineum* survival was the dependent variable.

### Is the fungal/AMF community changing across invasion time?

Changes in the general fungal community on *M*. *vimineum* roots were associated with time since invasion (P = 0.040, R^2^ = 0.1481) and % canopy openness (P = 0.021, R^2^ = 0.1615). When we included time since invasion in the model with % canopy openness, time since invasion maintained its significant association with the general fungal community composition on *M*. *vimineum* roots (P = 0.022, R^2^ = 0.1472). Changes in the root AMF community were not associated with time since invasion (P = 0.489, R^2^ = 0.0754).

## Discussion

Over time invasive species may lose the ecological advantages they gain due to their evolutionary novelty in a community [14–16]. We proposed that this phenomenon would manifest in population dynamics of the invader: namely, that over time invasive populations would develop stronger regulation, via more negative frequency dependence, and/or reduced ability to persist when rare. Understanding how population dynamics change as the population increases or decreases in frequency are crucial to predicting whether a species will come to dominate an area at the exclusion of other plant species, decline to local extinction in the community, or persist while allowing the coexistence of other species [21–22]. Our results support the latter prediction (reduced ability to persist when rare), but not the former (more negative frequency dependence), and suggest that over time *M*. *vimineum* populations may become more prone to local extinction. The decrease in survival at low frequency could not be explained by co-varying environmental gradients, but patterns in fungal communities on *M*. *vimineum* roots across time could be related to declines in survival.

Frequency dependence of survival shifted from negative to neutral with increasing time since invasion across our 12 sites. This change in frequency dependence across time since invasion was partially explained by other environmental variables. Contrary to our hypothesis, these results provide no evidence for accumulation of negative frequency dependence through invasion time. At recently invaded sites survival was negatively correlated with frequency of *M*. *vimineum*, suggesting negative frequency dependence. At older invaded sites the relationship was neutral with similar survival across all frequencies, suggesting neutral frequency dependence. However, this neutral pattern resulted from low survival across all frequencies. *Microstegium vimineum* seedling survival to reproduction is an important component of life-time fitness, affecting its recruitment ability and thus the per capita population growth rate [52]. Survival to reproduction is especially important at low *M*. *vimineum* frequency due to the necessity of having individuals present to produce seed and maintain the population. *Microstegium vimineum* survival at low frequency was negatively correlated with time since invasion, as we hypothesized. This suggests that *M*. *vimineum* per capita population growth rate at low frequency is declining through invasion time, decreasing its ability to persist when rare within a community [21, 22]. A population could compensate for declining survivorship if the per capita fecundity of the survivors increased, due to the release from intraspecific competition. However, we did not observe any evidence for this compensation when we analyzed the average living biomass as a proxy for fecundity [49]. Rather, the final biomass of surviving plants was unrelated to invasion history. This suggests that whatever is driving the change in the ability of *M*. *vimineum* to persist when rare is taking place at earlier stages of development, rather than during biomass accumulation.

Although the negative frequency dependence of *M*. *vimineum* survival at more recently invaded sites suggests coexistence between it and other plant community members, this does not rule out the possibility of it being a dominant invader. The recent populations still may be able to reach high frequency and be a dominant community member, while the other community members persist at low frequency. Due to the scope of our study we cannot determine this, but this study does suggest that *M*. *vimineum* has more potential to be a dominant community member at recently invaded sites compared to older invaded sites, where this is less likely due to lower survival rates across all frequencies.

The strength and direction of frequency dependence was also associated with latitude, soil nutrients, and % canopy openness. Generally, for the soil nutrients frequency dependence was neutral at low soil nutrients and then negative at high soil nutrients, driven mainly by a decline in survival rates at high frequencies in higher soil nutrients. Patterns of frequency dependence for soil nutrients may have resulted from greater self-thinning at more productive sites [53], since *M*. *vimineum* individual performance tends to positively respond to increased soil nutrients [54]. A decline in survival at high frequency may not harm *M*. *vimineum*’s population since conspecifics could compensate for the loss in seed production. Across low and high % canopy openness frequency dependence was more neutral due to similar increases in survival at both high and low frequencies as % canopy openness increased. Frequency dependence was neutral at low latitudes and negative at higher latitudes. This pattern was driven by a decrease in survival at high frequency at higher latitudes. A shift in the relative importance of intraspecific resource competition (leading to self-thinning at high frequency) relative to natural enemies (leading to low survival at all frequencies) from high to low latitudes could explain this pattern [55]. Unlike the decline in survival at low frequency with time since invasion these other environmental gradients show patterns in survival mainly at high frequency (see S2 Table), suggesting that there are different underlying processes for the pattern across time compared to the patterns across the other variables. Importantly, even when controlling for these environmental variables the pattern of decreasing survival with increasing time since invasion was maintained.

One possible mechanism for the decline in *M*. *vimineum*’s survival with invasion time is an accumulation of a more antagonistic soil community [16, 20]. We observed a change in the general fungal community on *M*. *vimineum* roots across invasion time but not specifically in the AMF community. Other fungal guilds in the root fungal community include pathogens, saprotrophs, and endophytes, and it is unclear which of these groups was driving fungal community changes across time. Although we cannot determine if the changes in fungal community composition across sites directly contributes to the reduced survival of *M*. *vimineum* individuals, the change in the *M*. *vimineum* root fungal community across invasion history is an interesting pattern and its connection to *M*. *vimineum* population dynamics through invasion history deserves future exploration. An alternative mechanism for a decline in an invader’s survival across invasion history could be an accumulation of aboveground enemies [18, 56]. However, we found minimal evidence, in the spring or fall, of aboveground herbivory/ pathogen infection on *M*. *vimineum* at the 12 sites used in this study (5.5% and 1.6% of all tagged individuals visually inspected in spring and fall respectively). Another possible explanation for the pattern is evolved improved competitive ability of the native plant community through time [57], which could be explored in future *M*. *vimineum* studies.

Our results suggest management of *M*. *vimineum* will be more successful at older invaded sites. Multiple management options are used against *M*. *vimineum* including herbicides and physical removal [58], all with the goal of local eradication (removal) or control. All options would be more effective at older invaded sites due to the decline in *M*. *vimineum* survival (and likely per capita population growth rate) at low frequency. At younger invaded sites, interventions that reduce *M*. *vimineum* populations to low frequency within the community may be counteracted by its high per capita population growth rate when rare. On the other hand, a reduction in *M*. *vimineum* populations may lead to local extinction due to low survival at low frequency at older invaded sites. Future studies that examine *M*. *vimineum* control across time since invasion could benefit management efforts.

## Conclusion

Our results are consistent with other studies that suggest the invasive advantage, gained from lacking coevolutionary history with the invaded community, can degrade through invasion time [14–16]. Our data demonstrate changes in the relationship between survivorship and frequency for *M*. *vimineum* over a scale of decades, that would benefit native plant species in the community. Although our study does not define a clear mechanism for the decline in survivorship of *M*. *vimineum* across time, our data suggests that the belowground fungal community could be a driver, which is consistent with studies on other species [16, 18–20], and calls for further exploration of this potential mechanism. Our study adds to the current literature by focusing on the invader’s key vital rates across invasion time, which will ultimately determine population dynamics and persistence. Understanding how an invader’s population dynamics change through invasion time is vital to predict its long-term impact and design the most effective management strategies.

## Supporting information

S1 Protocol Soil nutrient and root fungal community methods. (DOCX)

S1 Table Loadings of the 12 soil nutrient variables on the 12 PCA axes. (DOCX)

S2 Table Statistical results for general linear models testing survival in only low or high frequency plots across multiple variables. Statistics for general linear models with binomial distributions and proportion of *Microstegium vimineum* survivors as the dependent variable using only the highest or lowest frequency (freq) plots from each site, n = 12 for each subset (except for soil nutrient PC axes 1–3 n = 11). * = significant at the p≤0.1 level, ** = significant at the p≤0.05 level, *** = significant at the p≤0.001 level; time = time since invasion, PC1-3 = soil nutrient PC axes 1–3, canopy = % canopy openness.
(DOCX)

S3 Table Correlation coefficients of the model terms included in the full model for stepwise AIC. Correlation coefficients of the model terms included in the full model for stepwise AIC (n = 77). The probability of *M*. *vimineum* survival was the dependent variable. Freq = *M*. *vimineum* frequency, time = time since *M*. *vimineum* invasion, PC1- PC3 = soil nutrient PCA axes 1–3, canopy open = % canopy openness.
(DOCX)

S1 Dataset Data used for the statistical analyses of *M*. *vimineum* biomass and survival. (XLSX)

S2 Dataset Data used for the statistical analyses of the *M*. *vimineum* root general fungal community. (XLSX)

S3 Dataset Data used for the statistical analyses of the *M*. *vimineum* root arbuscular mycorrhizal fungal community. (XLSX)

S4 Dataset Database of *M*. *vimineum* collections retrieved from herbariums throughout the eastern United States. (XLSX)

## References

[1] Hallett SG. Dislocation from coevolved relationships: a unifying theory for plant invasion and naturalization? Weed Science. 2006;54(2):282–90.

[2] Hierro JL, Callaway RM. Allelopathy and exotic plant invasion. Plant and Soil. 2003;256(1):29–39. doi: 10.1023/a:1026208327014

[3] Callaway RM, Ridenour WM. Novel weapons: invasive success and the evolution of increased competitive ability. Frontiers in Ecology and the Environment. 2004;2(8):436–43. doi: 10.1890/1540-9295(2004)002[0436:nwisat]2.0.co;2

[4] Zheng YL, Feng YL, Zhang LK, Callaway RM, Valiente-Banuet A, Luo DQ, et al Integrating novel chemical weapons and evolutionarily increased competitive ability in success of a tropical invader. New Phytologist. 2015;205(3):1350–9. doi: 10.1111/nph.13135 25367824 10.1111/nph.13135

[5] Mitchell CE, Power AG. Release of invasive plants from fungal and viral pathogens. Nature. 2003;421(6923):625–7. doi: 10.1038/nature01317 12571594 10.1038/nature01317

[6] DeWalt SJ, Denslow JS, Ickes K. Natural-enemy release facilitates habitat expansion of the invasive tropical shrub Clidemia hirta. Ecology. 2004;85(2):471–83. doi: 10.1890/02-0728

[7] Liu H, Stiling P. Testing the enemy release hypothesis: a review and meta-analysis. Biological Invasions. 2006;8(7):1535–45. doi: 10.1007/s10530-005-5845-y

[8] Lloret F, Medail F, Brundu G, Camarda I, Moragues E, Rita J, et al Species attributes and invasion success by alien plants on Mediterranean islands. Journal of Ecology. 2005;93(3):512–20. doi: 10.1111/j.1365-2745.2005.00979.x

[9] Brym ZT, Lake JK, Allen D, Ostling A. Plant functional traits suggest novel ecological strategy for an invasive shrub in an understorey woody plant community. Journal of Applied Ecology. 2011;48(5):1098–106. doi: 10.1111/j.1365-2664.2011.02049.x

[10] Godoy O, Castro-Diez P, Valladares F, Costa-Tenorio M. Different flowering phenology of alien invasive species in Spain: evidence for the use of an empty temporal niche? Plant Biology. 2009;11(6):803–11. doi: 10.1111/j.1438-8677.2008.00185.x 19796357 10.1111/j.1438-8677.2008.00185.x

[11] Vila M, Weiner J. Are invasive plant species better competitors than native plant species? evidence from pair-wise experiments. Oikos. 2004;105(2):229–38. doi: 10.1111/j.0030-1299.2004.12682.x

[12] Vila M, Espinar JL, Hejda M, Hulme PE, Jarosik V, Maron JL, et al Ecological impacts of invasive alien plants: a meta-analysis of their effects on species, communities and ecosystems. Ecology Letters. 2011;14(7):702–8. doi: 10.1111/j.1461-0248.2011.01628.x 21592274 10.1111/j.1461-0248.2011.01628.x

[13] Sax DF, Gaines SD, Brown JH. Species invasions exceed extinctions on islands worldwide: A comparative study of plants and birds. American Naturalist. 2002;160(6):766–83. ISI:000179809400007. doi: 10.1086/343877 18707464 10.1086/343877

[14] Lankau RA, Nuzzo V, Spyreas G, Davis AS. Evolutionary limits ameliorate the negative impact of an invasive plant. Proceedings of the National Academy of Sciences of the United States of America. 2009;106(36):15362–7. doi: 10.1073/pnas.0905446106 19706431 10.1073/pnas.0905446106 PMC2730356

[15] Iacarella JC, Mankiewicz PS, Ricciardi A. Negative competitive effects of invasive plants change with time since invasion. Ecosphere. 2015;6(7):14 doi: 10.1890/es15-00147.1

[16] Dostal P, Muellerova J, Pysek P, Pergl J, Klinerova T. The impact of an invasive plant changes over time. Ecology Letters. 2013;16(10):1277–84. doi: 10.1111/ele.12166 23953187 10.1111/ele.12166

[17] Flory SL, Clay K. Pathogen accumulation and long-term dynamics of plant invasions. Journal of Ecology. 2013;101(3):607–13. doi: 10.1111/1365-2745.12078

[18] Mitchell CE, Blumenthal D, Jarosik V, Puckett EE, Pysek P. Controls on pathogen species richness in plants' introduced and native ranges: roles of residence time, range size and host traits. Ecol Lett. 2010;13(12):1525–35. doi: 10.1111/j.1461-0248.2010.01543.x 20973907 10.1111/j.1461-0248.2010.01543.x PMC3003901

[19] Hawkes CV. Are invaders moving targets? The generality and persistence of advantages in size, reproduction, and enemy release in invasive plant species with time since introduction. American Naturalist. 2007;170(6):832–43. doi: 10.1086/522842 18171166 10.1086/522842

[20] Diez JM, Dickie I, Edwards G, Hulme PE, Sullivan JJ, Duncan RP. Negative soil feedbacks accumulate over time for non-native plant species. Ecology Letters. 2010;13(7):803–9. doi: 10.1111/j.1461-0248.2010.01474.x 20482584 10.1111/j.1461-0248.2010.01474.x

[21] Adler PB, HilleRisLambers J, Levine JM. A niche for neutrality. Ecology Letters. 2007;10(2):95–104. doi: 10.1111/j.1461-0248.2006.00996.x 17257097 10.1111/j.1461-0248.2006.00996.x

[22] Chesson P. Mechanisms of maintenance of species diversity. Annual Review of Ecology and Systematics. 2000;31:343–66. doi: 10.1146/annurev.ecolsys.31.1.343

[23] Burdon JJ, Chilvers GA. Host density as a factor in plant-disease ecology. Annual Review of Phytopathology. 1982;20:143–66. doi: 10.1146/annurev.py.20.090182.001043

[24] Clarke B. Evidence for apostatic selection. Heredity. 1969;24:347–52. doi: 10.1038/hdy.1969.52 5262945 10.1038/hdy.1969.52

[25] May RM, Anderson RM. Epidemiology and genetics in the coevolution of parasites and hosts. Proceedings of the Royal Society Series B-Biological Sciences. 1983;219(1216):281–313. doi: 10.1098/rspb.1983.0075 10.1098/rspb.1983.0075 6139816

[26] Flory SL, Kleczewski N, Clay K. Ecological consequences of pathogen accumulation on an invasive grass. Ecosphere. 2011;2(10). doi: 10.1890/es11-00191.1

[27] Fairbrothers DE, Gray JR. Microstegium vimineum (Trin.) A. Camus (Gramineae) in the United States. Bulletin of the Torrey Botanical Club. 1972;99(2):97–100. doi: 10.2307/2484205

[28] Winter K, Schmitt MR, Edwards GE. Microstegium vimineum, a shade adapted C4 grass. Plant Science Letters. 1982;24(3):311–8. doi: 10.1016/0304-4211(82)90027-x

[29] Horton JL, Neufeld HS. Photosynthetic responses of Microstegium vimineum (Trin.) A. Camus, a shade-tolerant, C-4 grass, to variable light environments. Oecologia. 1998;114(1):11–9. doi: 10.1007/s004420050414 28307549 10.1007/s004420050414

[30] Flory SL, Clay K. Non-native grass invasion alters native plant composition in experimental communities. Biological Invasions. 2010;12(5):1285–94. doi: 10.1007/s10530-009-9546-9

[31] Oswalt CM, Oswalt SN, Clatterbuck WK. Effects of Microstegium Vimineum (Trin.) A. Camus on native woody species density and diversity in a productive mixed-hardwood forest in Tennessee. Forest Ecology and Management. 2007;242(2–3):727–32. doi: 10.1016/j.foreco.2007.02.008

[32] Adams SN, Engelhardt KAM. Diversity declines in Microstegium vimineum (Japanese stiltgrass) patches. Biological Conservation. 2009;142(5):1003–10. doi: 10.1016/j.biocon.2009.01.009

[33] Flory SL, Clay K. Non-native grass invasion suppresses forest succession. Oecologia. 2010;164(4):1029–38. doi: 10.1007/s00442-010-1697-y 20582439 10.1007/s00442-010-1697-y

[34] Marshall JM, Buckley DS, Franklin JA. Competitive interaction between Microstegium vimineum and first-year seedlings of three central hardwoods. Journal of the Torrey Botanical Society. 2009;136(3):342–9. doi: 10.3159/09-ra-006.1

[35] Strickland MS, DeVore JL, Maerz JC, Bradford MA. Loss of faster-cycling soil carbon pools following grass invasion across multiple forest sites. Soil Biology & Biochemistry. 2011;43(2):452–4. doi: 10.1016/j.soilbio.2010.10.006

[36] Fraterrigo JM, Strickland MS, Keiser AD, Bradford MA. Nitrogen uptake and preference in a forest understory following invasion by an exotic grass. Oecologia. 2011;167(3):781–91. doi: 10.1007/s00442-011-2030-0 21625979 10.1007/s00442-011-2030-0

[37] Kourtev PS, Ehrenfeld JG, Haggblom M. Experimental analysis of the effect of exotic and native plant species on the structure and function of soil microbial communities. Soil Biology & Biochemistry. 2003;35(7):895–905. doi: 10.1016/s0038-0717(03)00120-2

[38] DeMeester JE, Richter DD. Differences in wetland nitrogen cycling between the invasive grass Microstegium vimineum and a diverse plant community. Ecological Applications. 2010;20(3):609–19. doi: 10.1890/09-0283.1 20437951 10.1890/09-0283.1

[39] Flory SL, Long FR, Clay K. Invasive Microstegium populations consistently outperform native range populations across diverse environments. Ecology. 2011;92(12):2248–57. 22352164 10.1890/11-0363.1

[40] Ehrenfeld JG, Kourtev P, Huang WZ. Changes in soil functions following invasions of exotic understory plants in deciduous forests. Ecol Appl. 2001;11(5):1287–300. doi: 10.2307/3060920

[41] Lee MR, Flory SL, Phillips RP. Positive feedbacks to growth of an invasive grass through alteration of nitrogen cycling. Oecologia. 2012;170(2):457–65. doi: 10.1007/s00442-012-2309-9 22526935 10.1007/s00442-012-2309-9

[42] Glasgow LS, Matlack GR. The effects of prescribed burning and canopy openness on establishment of two non-native plant species in a deciduous forest, southeast Ohio, USA. For Ecol Manage. 2007;238(1–3):319–29. doi: 10.1016/j.foreco.2006.10.025

[43] Kuebbing S, Rodriguez-Cabal MA, Fowler D, Breza L, Schweitzer JA, Bailey JK. Resource availability and plant diversity explain patterns of invasion of an exotic grass. J Plant Ecol. 2013;6(2):141–9. doi: 10.1093/jpe/rts018

[44] Eschtruth AK, Battles JJ. Assessing the relative importance of disturbance, herbivory, diversity, and propagule pressure in exotic plant invasion. Ecological Monographs. 2009;79(2):265–80. doi: 10.1890/08-0221.1

[45] Environmental Systems Research Institute. ArcGIS Desktop Help 10.1 http://resources.arcgis.com/en/help/main/10.1/index.html2013 [cited 2014].

[46] Valeriano MD, Rossetti DD. Topodata: Brazilian full coverage refinement of SRTM data. Applied Geography. 2012;32(2):300–9. doi: 10.1016/j.apgeog.2011.05.004

[47] Frazer GW, Canham CD, Lertzman KP. Gap light analyzer (GLA), version 2.0: imaging software to extract canopy structure and gap light transmission indices from true-colour fisheye photographs, users manual and program documentation Burnaby, British Columbia and the Institute of Ecosystem Studies, Millbrook, NY Simon Fraser University; 1999.

[48] R Core Team. R: A Language and Environment for Statistical Computing. 2016.

[49] Wilson CH, Caughlin TT, Civitello DJ, Flory SL. Combining mesocosm and field experiments to predict invasive plant performance: a hierarchical Bayesian approach. Ecology. 2015;96(4):1084–92. doi: 10.1890/14-0797.1 26230028 10.1890/14-0797.1

[50] Gibson DJ, Spyreas G, Benedict J. Life history of Microstegium vimineum (Poaceae), an invasive grass in southern Illinois. Journal of the Torrey Botanical Society. 2002;129(3):207–19. doi: 10.2307/3088771

[51] Adler PB, Ellner SP, Levine JM. Coexistence of perennial plants: an embarrassment of niches. Ecology Letters. 2010;13(8):1019–29. doi: 10.1111/j.1461-0248.2010.01496.x 20545729 10.1111/j.1461-0248.2010.01496.x

[52] Warren RJ II, Bahn V, Bradford MA. Decoupling litter barrier and soil moisture influences on the establishment of an invasive grass. Plant and Soil. 2013;367(1–2):339–46. doi: 10.1007/s11104-012-1477-z

[53] Morris EC. How does fertility of the substrate affect intraspecific competition? Evidence and synthesis from self-thinning. Ecol Res. 2003;18(3):287–305. doi: 10.1046/j.1440-1703.2003.00555.x

[54] Ross KA, Ehrenfeld JG, Patel MV. The effects of nitrogen addition on the growth of two exotic and two native forest understory plants. Biological Invasions. 2011;13(10):2203–16. doi: 10.1007/s10530-011-0034-7

[55] Schemske DW, Mittelbach GG, Cornell HV, Sobel JM, Roy K. Is There a Latitudinal Gradient in the Importance of Biotic Interactions? Annual Review of Ecology Evolution and Systematics. 2009;40:245–69. doi: 10.1146/annurev.ecolsys.39.110707.173430

[56] Stricker KB, Harmon PF, Goss EM, Clay K, Flory SL. Emergence and accumulation of novel pathogens suppress aninvasive species. Ecology Letters. 2016;19(4):469–77. doi: 10.1111/ele.12583 26931647 10.1111/ele.12583

[57] Lankau RA. Coevolution between invasive and native plants driven by chemical competition and soil biota. Proceedings of the National Academy of Sciences of the United States of America. 2012;109(28):11240–5. doi: 10.1073/pnas.1201343109 22733785 10.1073/pnas.1201343109 PMC3396473

[58] Ward JS, Mervosh TL. Nonchemical and Herbicide Treatments for Management of Japanese Stiltgrass (Microstegium vimineum). Invasive Plant Science and Management. 2012;5(1):9–19. doi: 10.1614/ipsm-11-00018.1

