# Supplementary material for: Declining survival across invasion history for Microstegium vimineum
Source: PLoS One. 2017 Aug 15;12(8):e0183107. doi: 10.1371/journal.pone.0183107 (PMC5557486; doi:10.1371/journal.pone.0183107)
Supplement: S1 Protocol — (DOCX) [file pone.0183107.s001.docx]

**S1 Protocol. Soil nutrient and root fungal community methods.**

**Soil nutrient methods**

We measured nitrate (NO_3_^-^) and ammonium (NH_4_^+^) in each plot by placing 20 g wet mass of mixed bed ion exchange resin (Rexyn^TM^ 300 (H-OH) Beads (Analytical Grade/ Certified), Fisher Chemical) in the field for the duration of the study (~ 4 months). Bags for the resin were made of nylon stockings cut into 10 cm squares and zip tied closed. We charged the resin bags with 0.5 M HCl and rinsed them with DI water until the pH was neutral. We used a putty knife to make an angled slit in the soil and placed the resin bag within the slit at a depth of ~10cm within each plot. We collected the bags during the fall sampling, rinsed them with DI water, and individually extracted them with 100 ml of 2M KCl. Extracts were analyzed for NO_3_^-^ and NH_4_^+^ with a continuous flow colorimetric assay at the Stable Isotope Ecology Laboratory at the University of Georgia (Athens, GA). Our final sample size was 78 due to loss of resin bags over the season.

During spring sampling, we collected soil from the top 10cm within each plot and oven dried it to analyze general nutrients. From each site we combined equal amounts of the 8 invaded plot samples (4 for BEF and CNF), resulting in 12 soil samples (1 per site) for processing. We had a routine nutrient test performed at the University of Georgia Soil, Plant, and Water Laboratory (Athens, GA). The test provided measurements of soil pH and extractable phosphorus (P), potassium (K), calcium (Ca), magnesium (Mg), manganese (Mn), and zinc (Zn). We also analyzed total % nitrogen (N) and carbon (C) and calculated the C:N ratio of the oven-dried soil. For the C and N analysis, we ball-milled the soil samples to less than 250 µm particle size and weighed 24-26 mg into 5 x 5 mm tin capsules. The capsules were analyzed with Micro-Dumas combustion at the Stable Isotope Ecology Laboratory at the University of Georgia (Athens, GA).

**Root fungal community methods**

During the spring sampling, we collected *M. vimineum* roots from each plot to perform terminal restriction fragment length polymorphism analysis (T-RFLP) of the general fungal and arbuscular mycorrhizal fungal (AMF) community. We collected fine roots from 2-5 *M. vimineum* individuals in each plot and pooled the roots for one sample per plot. We stored all the samples for molecular analysis on ice until returning to the lab where we stored them in a -80°C freezer.

We extracted DNA from all materials using Omega Bio-tek (Atlanta, GA) extraction kits. For the general fungal community, the polymerase chain reaction (PCR) targeted the internal transcribed spacer (ITS) region of the ribosomal RNA gene segment using primer pair ITSf1 and ITS4r (49, 50). For the AMF PCR, the small subunit ribosomal RNA region was targeted using primer pair AML1 and AML2 (51). The PCR protocols followed those in St. Laurent et al. (2008) for general fungi. The PCR protocols for AMF were from Lee et al. (2008), except 0.5g of T4 gene 32 protein (Roche Diagnostics) was added to the AMF PCR and PCR cycles were increased to 35. The forward primers were labelled with 6-FAM fluorescence (Operon Biotechnologies, Inc., Huntsville, AL, USA) to allow us to detect fragments with capillary electrophoresis.

After PCR, the products were digested with a restriction enzyme; Hha for general fungi and Mbo for AMF (Promega). Capillary electrophoresis on an ABI Prism 3730xl DNA analyzer (Applied Biosystems, Carlsbad, CA, USA) sized the fragments using a fluorescent lane standard, ROX1000. We then used GENEMAPPER v 3.0 (Applied Biosystems, Carlsbad, CA, USA) for size-calling of the fluorescence peaks using two basepair allele bins. Peak areas, roughly equivalent to the relative abundance of the operational taxonomic unit (OTU) represented by that fragment, were used for all analyses.
