# Supplementary material for: Declining survival across invasion history for Microstegium vimineum
Source: PLoS One. 2017 Aug 15;12(8):e0183107. doi: 10.1371/journal.pone.0183107 (PMC5557486; doi:10.1371/journal.pone.0183107)
Supplement: S1 Table — (DOCX) [file pone.0183107.s002.docx]

|  | PCA 1 | PCA 2 | PCA 3 | PCA 4 | PCA 5 | PCA 6 | PCA 7 | PCA 8 | PCA 9 | PCA 10 | PCA 11 | PCA 12 |
| --- | --- | --- | --- | --- | --- | --- | --- | --- | --- | --- | --- | --- |
| NO_3_^-^ | -0.2868 | 0.0489 | 0.4531 | 0.0606 | -0.1826 | -0.5870 | -0.2442 | 0.5059 | -0.0923 | -0.0553 | 0.0351 | -0.0045 |
| NH_4_^+^ | -0.2139 | -0.0318 | 0.3226 | 0.5824 | 0.6937 | 0.0854 | 0.1199 | -0.0688 | 0.0182 | -0.0299 | -0.0147 | -0.0295 |
| Total N | -0.4095 | 0.0946 | 0.1955 | -0.1578 | -0.1660 | 0.2251 | 0.1462 | -0.0908 | 0.0266 | -0.2224 | -0.7729 | 0.0698 |
| Total C | -0.3784 | 0.1614 | 0.1637 | -0.1762 | -0.1401 | 0.3975 | 0.3684 | 0.1322 | -0.2460 | -0.1637 | 0.5082 | -0.3106 |
| C:N | 0.2040 | -0.2149 | 0.4749 | -0.3804 | 0.2005 | 0.4446 | -0.3557 | 0.1802 | -0.1530 | 0.2966 | -0.0127 | 0.1833 |
| pH | 0.2836 | 0.3791 | 0.2380 | -0.0789 | 0.0676 | -0.1017 | -0.3522 | -0.4549 | -0.3160 | -0.4643 | 0.0173 | -0.2267 |
| Ca | 0.1517 | 0.4719 | 0.3565 | -0.0430 | -0.1008 | 0.0388 | 0.0833 | -0.0747 | 0.7286 | 0.2207 | 0.0846 | -0.1201 |
| K | -0.0501 | 0.5790 | -0.0492 | 0.0050 | 0.0505 | -0.1194 | 0.1949 | -0.0865 | -0.4481 | 0.5124 | -0.0447 | 0.3636 |
| Mg | 0.1059 | 0.4443 | -0.3747 | -0.0603 | 0.3270 | 0.1950 | -0.1707 | 0.6151 | 0.0796 | -0.2695 | -0.1240 | -0.0238 |
| Mn | -0.4004 | 0.0227 | -0.0842 | -0.3574 | 0.2466 | -0.1199 | -0.1426 | -0.2276 | 0.2581 | -0.2578 | 0.3130 | 0.5709 |
| P | -0.3078 | 0.1256 | -0.1371 | 0.4627 | -0.3561 | 0.3685 | -0.5936 | -0.0946 | 0.0301 | 0.1216 | 0.1050 | 0.0775 |
| Zn | -0.3838 | -0.0039 | -0.2183 | -0.3209 | 0.2936 | -0.1657 | -0.2676 | -0.1487 | 0.0331 | 0.3887 | -0.0942 | -0.5776 |

**S1 Table. Loadings of the 12 soil nutrient variables on the 12 PCA axes.**
