# Supplementary material for: Declining survival across invasion history for Microstegium vimineum
Source: PLoS One. 2017 Aug 15;12(8):e0183107. doi: 10.1371/journal.pone.0183107 (PMC5557486; doi:10.1371/journal.pone.0183107)
Supplement: S2 Table — Statistics for general linear models with binomial distributions and proportion of Microstegium vimineum survivors as the dependent variable using only the highest or lowest frequency (freq) plots from each site, n = 12 for each subset (except for soil nutrient PC axes 1–3 n = 11). * = significant at the p≤0.1 level, ** = significant at the p≤0.05 level, *** = significant at the p≤0.001 level; time = time since invasion, PC1-3 = soil nutrient PC axes 1–3, canopy = % canopy openness. (DOCX) [file pone.0183107.s003.docx]

**S2 Table.** **Statistical results for general linear models testing survival in only low or high frequency plots across multiple variables.**

|  | **low frequency plots** | | | | | **high frequency plots** | | | | |
| --- | --- | --- | --- | --- | --- | --- | --- | --- | --- | --- |
| **model term** | **estimate** | **standard error** | **LRT** | **p-value (LRT)** |  | **estimate** | **standard error** | **LRT** | **p-value (LRT)** |  |
| freq  time | 4.3797  -0.0766 | 3.2934  0.0346 | 1.8462  6.0360 | 0.1742  0.0140 | ** | 1.2070  0.0226 | 2.8772  0.0229 | 0.1789  0.9990 | 0.6723  0.3176 |  |
| freq  latitude | 0.5607  0.1794 | 2.9985  0.1477 | 0.0352  1.5204 | 0.8511  0.2176 |  | 2.9719  -0.3334 | 2.9529  0.1456 | 1.0404  5.7618 | 0.3077  0.0164 | ** |
| freq  PC1 | 0.8272  -0.1800 | 2.9683  0.1635 | 0.0786  1.5626 | 0.7792  0.2113 |  | 2.8567  0.3715 | 3.4068  0.2540 | 0.7236  3.2777 | 0.3950  0.0702 | * |
| freq  PC2 | 0.7823  -0.0047 | 3.0027  0.1677 | 0.0686  0.0008 | 0.7934  0.9775 |  | 1.0004  -0.3861 | 3.1903  0.1976 | 0.0996  4.6346 | 0.7523  0.0313 | ** |
| freq  PC3 | 0.7757  -0.0026 | 3.1199  0.2010 | 0.0627  0.0002 | 0.8022  0.9898 |  | -6.3631  -0.6418 | 4.3340  0.3634 | 2.2368  4.0326 | 0.1348  0.0446 | ** |
| freq  canopy | 3.5327  0.3975 | 3.8561  0.1646 | 0.9097  9.0504 | 0.3402  0.0026 | ** | 3.0655  0.0994 | 3.1659  0.0863 | 0.9507  1.3883 | 0.3295  0.2387 |  |

Statistics for general linear models with binomial distributions and proportion of *Microstegium vimineum* survivors as the dependent variable using only the highest or lowest *M. vimineum* frequency (freq) plots from each site, n=12 for each subset (except for soil nutrient PC axes 1-3 n=11). * = significant at the p≤0.1 level, ** = significant at the p≤0.05 level, *** = significant at the p≤0.001 level; time=time since invasion, PC1-3= soil nutrient PC axes 1-3, canopy=% canopy openness
