# Supplementary material for: Declining survival across invasion history for Microstegium vimineum
Source: PLoS One. 2017 Aug 15;12(8):e0183107. doi: 10.1371/journal.pone.0183107 (PMC5557486; doi:10.1371/journal.pone.0183107)
Supplement: S3 Table — Correlation coefficients of the model terms included in the full model for stepwise AIC (n = 77). The probability of M. vimineum survival was the dependent variable. Freq = M. vimineum frequency, time = time since M. vimineum invasion, PC1- PC3 = soil nutrient PCA axes 1–3, canopy open = % canopy openness. (DOCX) [file pone.0183107.s004.docx]

|  | freq | time | latitude | PC1 | PC2 | PC3 | canopy open |
| --- | --- | --- | --- | --- | --- | --- | --- |
| freq | 1.00 |  |  |  |  |  |  |
| time | 0.05 | 1.00 |  |  |  |  |  |
| latitude | 0.12 | -0.34 | 1.00 |  |  |  |  |
| PC1 | 0.04 | 0.38 | -0.37 | 1.00 |  |  |  |
| PC2 | 0.00 | -0.37 | 0.38 | 0.00 | 1.00 |  |  |
| PC3 | -0.12 | -0.20 | 0.22 | 0.00 | 0.00 | 1.00 |  |
| canopy open | 0.23 | 0.06 | 0.24 | 0.07 | 0.10 | 0.24 | 1.00 |

**S3 Table. Correlation coefficients of the model terms included in the full model for stepwise AIC.**

Correlation coefficients of the model terms included in the full model for stepwise AIC (n=77). The probability of *M. vimineum* survival was the dependent variable. Freq = *M. vimineum* frequency, time = time since *M. vimineum* invasion, PC1- PC3 = soil nutrient PCA axes 1-3, canopy open= % canopy openness
